# Triglyceride-Rich Lipoproteins and ASCVD: Evidence for Causality and Challenges in Therapeutic Translation

**DOI:** 10.1007/s11883-026-01442-y

**Published:** 2026-07-20

**Authors:** Elias Björnson, Martin Adiels

**Affiliations:** 1 Department of Molecular and Clinical Medicine, Institute of Medicine, University of Gothenburg, Gothenburg, Sweden; 2 School of Public Health and Community Medicine, Institute of Medicine, University of Gothenburg, Gothenburg, Sweden

**Keywords:** Triglycerides, Triglyceride-rich lipoproteins, TRL-C, ApoB, APOC3, Cardiovascular disease

## Abstract

**Purpose of Review:**

This review summarizes current evidence linking triglyceride-rich lipoproteins (TRLs) to atherosclerotic cardiovascular disease (ASCVD) with emphasis on challenges in translating the epidemiological- and genetic epidemiological findings into therapeutic risk reduction.

**Recent Findings:**

Elevated triglycerides and TRL-cholesterol are consistently associated with ASCVD risk in both primary- and secondary-prevention populations. Human genetic studies of pathways involving LPL, APOC3, ANGPTL3, and ANGPTL4 strongly support a causal role for TRL metabolism in atherosclerosis. The pathogenic effects of TRLs may differ from those of low-density lipoproteins (LDLs), with TRLs potentially contributing through both cholesterol deposition and activation of inflammatory pathways. However, therapeutic translation has been less straightforward than for LDL. Fibrates have produced inconsistent cardiovascular outcome results, icosapent ethyl reduces cardiovascular events but probably through mechanisms beyond triglyceride lowering alone, and potent APOC3 inhibition has failed to show short-term coronary plaque regression. Together, these findings suggest that TRLs are causal contributors to ASCVD, but that their therapeutic relevance may depend on disease stage, background LDL-C/apoB burden, and residual metabolic risk.

**Summary:**

TRLs are biologically and genetically linked to ASCVD, but whether lowering TRLs translates into cardiovascular risk reduction may depend on background therapy and cardiovascular risk context. Future trials must determine whether TRL-targeted therapies reduce ASCVD events beyond contemporary prevention, and in which patients this residual risk remains modifiable.

**Graphical Abstract:**

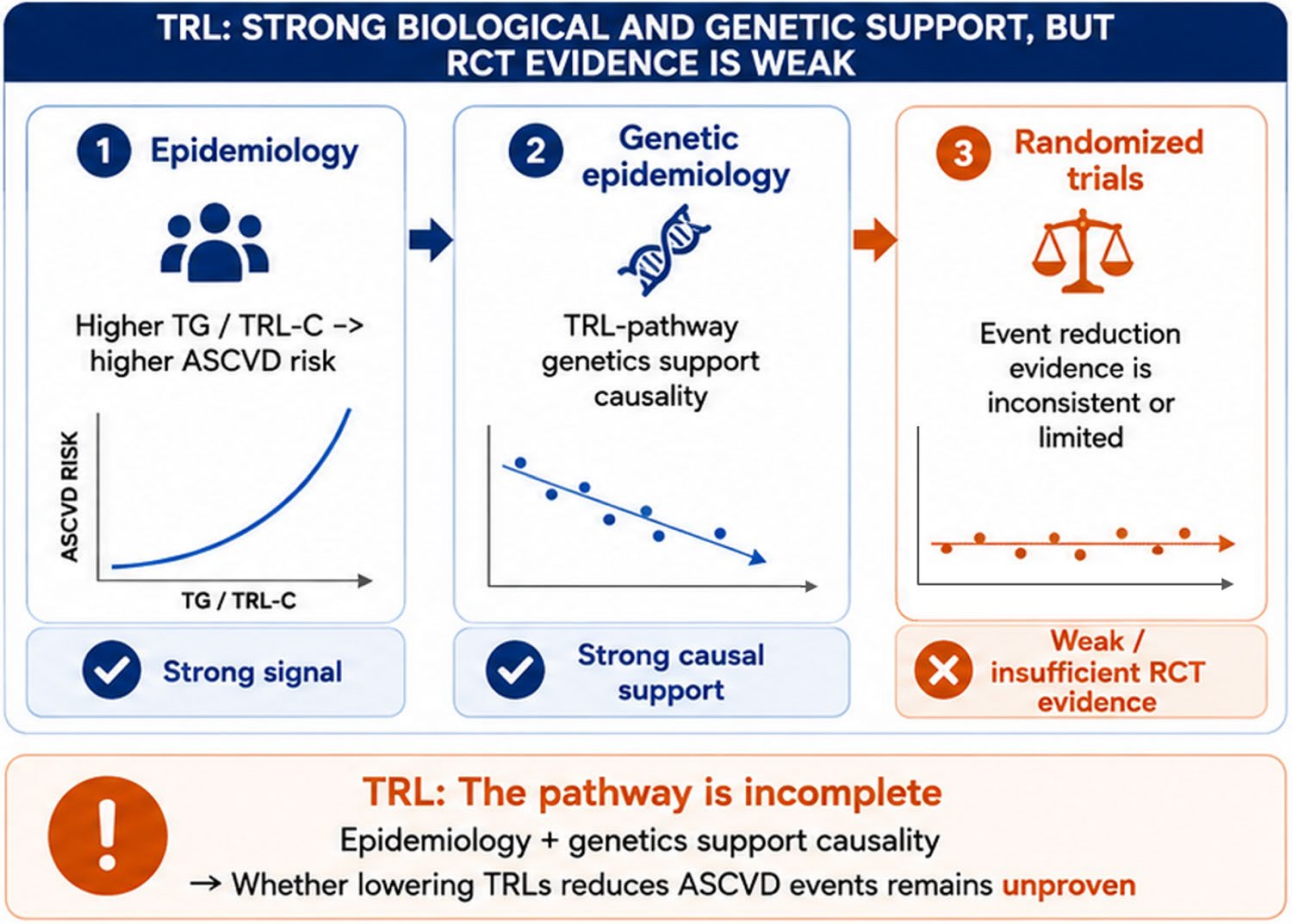

## Introduction

Our understanding of the role of triglyceride-rich lipoproteins (TRLs) in cardiovascular disease has followed a less linear path than that for low-density lipoproteins (LDLs). Epidemiological studies have linked higher LDL-cholesterol (LDL-C) to higher risk of atherosclerotic cardiovascular disease (ASCVD) [1, 2]. Human genetics and Mendelian randomization studies have demonstrated that lifelong lower LDL-C is associated with lower ASCVD risk [3–6]. Finally, numerous randomized trials have shown that LDL-C lowering reduces cardiovascular events in proportion to the absolute reduction in LDL-C, across statins, ezetimibe, PCSK9 inhibition, and other LDL-lowering strategies [7–12]. For LDL-C, the evidence all line up remarkably well.

Research around TRLs are moving along a similar path but have not reached its final destination. Epidemiological studies consistently show that elevated triglycerides and TRL-related lipid measures are associated with higher ASCVD risk [2]. Human genetic studies of pathways such as LPL, APOC3, ANGPTL3, and ANGPTL4 strongly suggest that TRL metabolism is causal [13–16]. However, the final step - demonstrating that pharmacological targeting of TRLs reliably reduces cardiovascular events - remains unsettled. Fibrates have produced inconsistent results, icosapent ethyl reduces events but may not act primarily through triglyceride lowering, and potent APOC3 inhibition has not yet shown coronary plaque regression in imaging studies [17–19].

This review asks how far the TRL hypothesis has progressed and whether it is likely to complete the journey. We argue that the question is no longer whether TRLs are biologically relevant to ASCVD. The stronger and more clinically useful question is whether TRLs can be lowered sufficiently, in the right patients and at the right disease stage, to produce cardiovascular risk reduction beyond contemporary LDL-C and apoB lowering.

## Epidemiological Evidence

TRLs are the collective name for the lipoproteins rich in triglycerides; chylomicrons, chylomicron remnants and very low-density lipoproteins. These particles are larger in size than the low-density lipoproteins because of their bulk triglyceride content. Measured plasma triglycerides comprise triglycerides contained in all lipoproteins, of which around 80% are in the TRLs. The TRLs also contain some cholesterol, referred to as TRL-cholesterol (TRL-C).

Elevated triglycerides are often part of a broader metabolic phenotype including obesity, insulin resistance, diabetes, low HDL-C, and small dense LDL particles. This has historically made it uncertain whether TRLs were directly atherogenic or simply markers of adverse metabolic health.

A large body of epidemiological evidence links elevated triglycerides and TRL-C measures to ASCVD. Although individual studies differ in whether triglycerides remain independently associated with risk after extensive adjustment for HDL-C, non-HDL-C, apoB, diabetes, adiposity, and other metabolic traits, the overall pattern is consistent: individuals with higher triglyceride concentrations have higher rates of coronary heart disease, ischemic stroke, cardiovascular mortality, and residual cardiovascular risk.

Early prospective data from the Framingham Study helped establish that triglycerides were associated with coronary heart disease, particularly in women and in individuals with the combined high-triglyceride/low-HDL-C phenotype [20–22]. A 1996 meta-analysis by Hokanson and Austin of population-based prospective studies found that triglycerides were associated with cardiovascular disease independently of HDL-C, with stronger associations in women than in men [23]. Subsequent pooled analyses confirmed this association across larger populations. In 29 Western prospective studies including over 10,000 incident coronary heart disease cases among over 250,000 participants, Sarwar et al. found that triglycerides were associated with coronary heart disease risk, although the association was attenuated after adjustment for HDL-C and other lipids [24]. A major individual-participant meta-analysis from the Emerging Risk Factors Collaboration, including more than 300,000 participants, similarly found that triglycerides were associated with coronary heart disease, but that the association was substantially weakened after adjustment for HDL-C and non-HDL-C [2].

The association is not restricted to Western populations. In the Asia Pacific Cohort Studies Collaboration, serum triglycerides were associated with both coronary heart disease and stroke risk, supporting the generalizability of the triglyceride-risk association across populations with different lipid distributions and cardiovascular risk profiles [25]. In the Atherosclerosis Risk in Communities study, triglycerides and other lipid-related measures were associated with incident coronary heart disease, although the apparent strength of different markers depended on model specification and adjustment for correlated lipid traits [26].

In the Copenhagen City Heart Study, elevated non-fasting triglycerides were associated with increased risk of myocardial infarction, ischemic heart disease, and death in both men and women [27]. In the Women’s Health Study, non-fasting triglycerides were more strongly associated with incident cardiovascular events than fasting triglycerides, independent of traditional risk factors and other lipids [28].

The association also extends to ischemic stroke. In the Copenhagen City Heart Study, non-fasting triglycerides showed a graded association with ischemic stroke over long-term follow-up [29]. A later Copenhagen analysis comparing non-fasting triglycerides and cholesterol also found that stepwise increases in non-fasting triglycerides were associated with ischemic stroke risk [30]. These findings suggest that elevated TRLs may not be limited to coronary disease, although the strength and consistency of associations may differ across vascular beds.

In the Copenhagen cohorts, higher calculated TRL-C was associated with higher risk of ischemic heart disease and myocardial infarction [31, 32]. In the Copenhagen General Population Study, elevated TRL-C was associated with myocardial infarction risk across normal-weight, overweight, and obese individuals, suggesting that TRL-C is not merely a marker of adiposity [33]. In individuals with ischemic heart disease, higher TRL-C also explained part of the residual risk of all-cause mortality, highlighting its relevance in secondary-prevention populations [34].

Meta-analytic evidence further supports the epidemiological association. A systematic review and meta-analysis of 61 prospective studies, including more than 700,000 participants for cardiovascular mortality analyses, found that higher triglycerides were associated with higher cardiovascular death and all-cause mortality; each 1 mmol/L increase in triglycerides was associated with a 13% higher risk of cardiovascular death [35].

Taken together, the epidemiological evidence supports two conclusions. First, elevated triglycerides are a robust marker of increased ASCVD risk across multiple cohorts, outcomes, and populations. Second, the strength of the association is attenuated, but not eliminated when adjusting for other co-variates indicating that TRLs are often elevated alongside other risk factors but appear nevertheless itself to be an independent risk factor for cardiovascular disease.

## Human Genetics and Mendelian Randomization: Evidence for Causality

For many years, the causal relevance of TRLs in atherosclerosis was difficult to distinguish from the broader metabolic phenotype. This made it uncertain whether TRLs were directly atherogenic or simply markers of adverse metabolic health. Human genetic epidemiology has helped resolve this uncertainty. By utilizing genetic variants that influence lipid biology from birth and are uncorrelated with other metabolic traits, Mendelian randomization has provided a powerful framework for testing whether TRLs contribute causally to ASCVD.

Early genome-wide and Mendelian randomization studies showed that TRL-associated variants were also associated with coronary artery disease risk. In one influential early analysis, Do et al. used common variants associated with plasma triglycerides and found that the magnitude of a variant’s triglyceride association correlated with its association with coronary artery disease, including in models accounting for LDL-C and HDL-C [36].

Another strong line of evidence has come from APOC3. ApoC-III inhibits lipoprotein lipase activity and impairs hepatic clearance of TRLs and their remnants. Rare loss-of-function variants in APOC3 are associated with markedly lower triglyceride levels and substantially lower coronary disease risk. In a large sequencing study, carriers of APOC3 loss-of-function variants had lower triglycerides and approximately 40% lower risk of coronary heart disease [14]. In parallel, Danish population studies showed that APOC3 loss-of-function variants were associated with lower non-fasting triglycerides and reduced risk of ischemic vascular disease and ischemic heart disease [37]. These studies were important because they linked a clearly interpretable biological mechanism—reduced apoC-III and subsequent enhanced TRL clearance—to lower ASCVD risk in humans.

Additional support comes from the angiopoietin-like proteins. ANGPTL4 and ANGPTL3 inhibit lipoprotein lipase and thereby modulate TRL metabolism. Inactivating variants in ANGPTL4 are associated with lower triglyceride concentrations and lower coronary artery disease risk [16]. Loss-of-function variants in ANGPTL3 are also associated with lower triglycerides and lower coronary disease risk, although interpretation is less specific because ANGPTL3 inhibition lowers multiple lipid fractions, including LDL-C [15]. Together, the genetic evidence supports the concept that lifelong differences in TRL metabolism and clearance influence atherosclerotic risk.

A more recent question is whether all apoB-containing lipoproteins have the same atherogenicity per particle. ApoB remains a central marker because each LDL, very-low-density lipoprotein, intermediate-density lipoprotein, and Lp(a) particle carries one apoB molecule. However, genetic analyses suggest that particle number may not be the entire story. One previous analysis have found similar coronary risk per unit change in apoB for LPL- and LDLR-mediated lipid lowering [38], whereas our group and others have shown, indirectly or directly, that TRLs is likely more atherogenic than LDL on a per-particle basis [39–42].

These genetic studies provide strong evidence for causality, but they do not by themselves prove that short-term pharmacological TRL lowering will reduce cardiovascular events. Genetic variants act throughout life, alter cumulative exposure over decades, and may influence several linked aspects of TRL biology at the same time. Drug trials usually intervene later, often in patients with established atherosclerosis, background statin therapy, and multiple competing risk factors. Thus, the central translational question is no longer whether TRL metabolism is causally related to ASCVD, but whether pharmacological intervention can reproduce the protective biology seen in genetic epidemiology studies with sufficient magnitude, duration, and specificity to reduce events.

## Mechanisms of TRL Atherogenicity

**Fig. 1:**
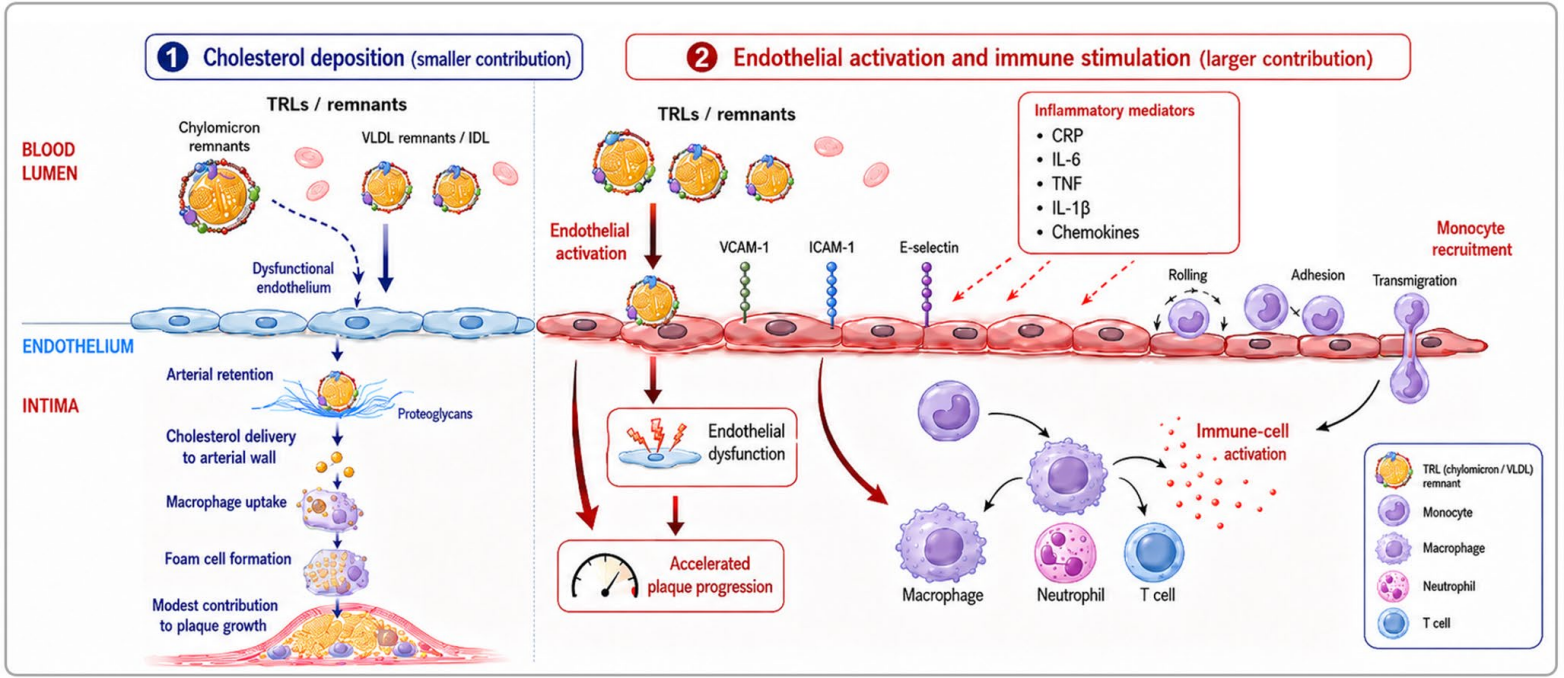
Proposed dual mechanisms of triglyceride-rich lipoproteins role in atherosclerosis. TRLs and their remnants may promote atherosclerosis through two partly overlapping pathways. First, TRLs can enter the arterial wall, bind to intimal proteoglycans, deliver cholesterol, and contribute to macrophage lipid accumulation and foam-cell formation. This pathway may add to plaque growth, although the cholesterol contribution of TRLs is considerably smaller than that of LDL particles. Second, TRLs may act as inflammatory accelerants by activating endothelial cells and stimulating immune-cell recruitment. Endothelial activation is characterized by increased expression of adhesion molecules such as VCAM-1, ICAM-1, and E-selectin, together with release of inflammatory mediators including CRP, IL-6, TNF, IL-1β, and chemokines. These processes promote monocyte rolling, adhesion, and transmigration, followed by activation of macrophages, neutrophils, and T cells within the vascular wall. Thus, TRLs may contribute to atherogenesis not only by delivering cholesterol to the intima, but also by amplifying endothelial dysfunction, vascular inflammation, and accelerated plaque progression. In this model, LDLs may contribute the majority of cholesterol-deposition but not contribute to the direct immune-stimulating action, hence the major role of TRLs may be to accelerate the predominantly LDL-driven plaque growth rather than to drive bulk cholesterol deposition alone.

To understand whether TRL-lowering agents may or may not reduce cardiovascular events in a clinical context it is important to understand the mechanism of TRLs atherogenic potential. Here, we propose that TRLs produce atherosclerosis by dual mechanisms: (1) cholesterol deposition and (2) inflammation and endothelial activation (Fig. 1).

### Cholesterol Deposition

TRLs can contribute to atherosclerosis by delivering cholesterol to the arterial wall. Smaller TRLs can enter the intima, become retained, and contribute to macrophage lipid loading. This mechanism fits the general apoB paradigm: apoB-containing particles are atherogenic when they enter and are retained in the arterial wall [43, 44]. TRL-C is important because it reflects cholesterol carried by TRLs capable of participating in plaque formation, whereas triglycerides themselves are not likely to contribute to the atherogenic process. TRLs typically contain more (1.5–3 times more) cholesterol per particle [45] and typically make up 10–20% of the non-HDL-C content. Therefore, it is likely that TRLs have a similar role to play as LDL-C does in cholesterol deposition, albeit to a considerably lower degree than LDL-C because of its lower concentration.

### Inflammation and Endothelial Activation

TRLs may also promote atherosclerosis through inflammatory mechanisms. Experimental and human data link TRL-rich states with endothelial dysfunction, adhesion molecule expression, monocyte recruitment, macrophage activation, and low-grade systemic inflammation [46–48]. Our group recently showed that TRLs causally increase plasma levels of not only C-reactive protein, but also neutrophils, white blood cells [42] and circulating chemokines [49]. Taken together, the emerging evidence in this area suggests that TRLs, but not LDLs, promote an inflammatory component in atherosclerosis.

### A Dual-Hit Model

A useful hypothesis or working model is therefore that TRLs have two related but distinct atherogenic effects. First, they deliver cholesterol to the artery wall and contribute to cholesterol deposition. Second, they accelerate plaque development through inflammatory and endothelial effects. The second mechanism may be most important in the presence of a permissive background of LDL-C or apoB exposure. In this framework, LDL provides much of the substrate for plaque growth, while TRLs accelerate progression or destabilization in metabolically adverse states.

This model may help explain why TRL-associated risk is strong in epidemiology and genetics but difficult to modify pharmacologically. If TRLs contribute to decades-long plaque initiation and acceleration, late intervention may not rapidly reverse established plaque. If TRLs amplify LDL-driven biology, their incremental importance may decline when LDL-C and apoB are already aggressively lowered. And if TRLs influence inflammation or thrombosis more than plaque volume, standard imaging endpoints may underestimate their clinical relevance.

## Therapeutic Evidence: Why Triglyceride Lowering Has Not Yet Mirrored LDL-C Lowering

### Fibrates

Fibrates provide the longest-standing pharmacological test of triglyceride lowering. Early trials were encouraging. The Helsinki Heart Study showed that gemfibrozil reduced coronary events in dyslipidemic men [50], and VA-HIT showed that gemfibrozil reduced cardiovascular events in men with coronary disease and low HDL-C [51]. Later trials were less definitive. In the FIELD study, in patients with type 2 diabetes, fenofibrate did not significantly reduce the primary coronary outcome, although some secondary outcomes were favorable [52]. ACCORD-Lipid, testing fenofibrate added to statin therapy in type 2 diabetes, was neutral overall, although a subgroup with high triglycerides and low HDL-C appeared to benefit [53].

PROMINENT was the most contemporary and stringent fibrate test. Pemafibrate lowered triglycerides, VLDL cholesterol, TRL-C and apoC-III in statin-treated patients with type 2 diabetes, elevated triglycerides, and low HDL-C, but did lead to an increase in LDL-C and did not reduce cardiovascular events [17]. PROMINENT is therefore a central cautionary trial: lowering triglyceride concentration, even in a seemingly appropriate population, may be insufficient if the intervention does not meaningfully reduce the relevant apoB-containing particle burden.

The fibrate story should therefore be interpreted cautiously. It does not refute TRL causality, but it argues against the simple proposition that any pharmacological reduction in triglyceride concentration will reduce ASCVD events.

### Omega-3 Fatty Acids and Icosapent Ethyl

Omega-3 fatty acid trials have also been heterogeneous. Trials of mixed EPA/DHA formulations have generally been neutral. ORIGIN did not reduce cardiovascular events in patients with dysglycemia [54], ASCEND was neutral in patients with diabetes without known cardiovascular disease [55], and STRENGTH was stopped for futility despite enrolling statin-treated high-risk patients with high triglycerides and low HDL-C [56].

REDUCE-IT was different. In statin-treated patients with elevated triglycerides and either established cardiovascular disease or diabetes plus risk factors, icosapent ethyl reduced the primary composite cardiovascular endpoint by 25% [18]. EVAPORATE, a coronary CT angiography study, suggested that icosapent ethyl slowed progression of adverse plaque features, supporting plaque-level effects beyond simple triglyceride lowering [57].

In a 2019 meta-analysis summing up all available evidence, omega-3 supplementation, whether or not REDUCE-IT was included or excluded from the analysis, lowered the risk for ASCVD events [58]. Thus, although there is heterogeneity in the literature, collected evidence clearly show cardiovascular benefit.

Taken together, omega-3 supplementation and in particular icosapent ethyl can be viewed as a therapy that mitigates TRL-associated risk, but not as definitive proof that lowering triglycerides themselves reduces events.

## Emerging Therapies Targeting TRL Metabolism

### APOC3 Inhibition

APOC3 inhibition is one of the most compelling approaches for TRL reduction because genetic, mechanistic, and pharmacological evidence align. ApoC-III delays TRL clearance and is strongly linked to hypertriglyceridemia. Volanesorsen, an antisense oligonucleotide targeting APOC3, markedly lowered triglycerides in familial chylomicronemia syndrome [59].

Newer agents appear more potent and better tolerated. Olezarsen, a GalNAc-conjugated antisense therapy targeting APOC3, reduced triglycerides substantially in patients with moderate hypertriglyceridemia and elevated cardiovascular risk in moderate hypertriglyceridemic individuals [60]. Plozasiran, an siRNA targeting APOC3, reduced triglycerides in both mixed hyperlipidemia and severe hypertriglyceridemia [61, 62]. These therapies may transform treatment of hypertriglyceridemia and powerfully reduce pancreatitis risk [63], but their role in ASCVD prevention remains unresolved.

### ANGPTL3 Inhibition

ANGPTL3 inhibition is also promising but mechanistically broader. ANGPTL3 regulates lipoprotein lipase and endothelial lipase, and its inhibition can lower TRLs and LDLs. Evinacumab, a monoclonal antibody targeting ANGPTL3, lowers LDL-C and triglycerides and is approved for homozygous familial hypercholesterolemia [64, 65].

Vupanorsen, an antisense therapy targeting ANGPTL3, lowered non-HDL-C and triglycerides in TRANSLATE-TIMI 70, but development was discontinued after concerns including liver fat and liver enzyme elevations [66]. Newer siRNA approaches may overcome some limitations, but outcome evidence is still lacking. Because ANGPTL3 affects several lipid fractions, any future event benefit may be more difficult to attribute specifically to TRL lowering.

## The ESSENCE-TIMI 73b Imaging Study: A Test Case for the TRL Hypothesis

The recent ESSENCE-TIMI 73b coronary CT angiography (CCTA) imaging study deserves separate attention because it crystallizes the central dilemma of the field. APOC3 inhibition is among the most genetically supported approaches to lowering TRLs. If any triglyceride-targeting therapy should plausibly modify atherosclerosis, an effective APOC3 inhibitor is high on the list. Olezarsen lowers triglycerides substantially, reduces TRL-C-related measures, and targets a pathway strongly supported by human genetics.

Yet, repeated CCTA did not show significant coronary plaque regression over 12 months. In the CCTA substudy, olezarsen reduced triglycerides by approximately 64%, TRL-C by approximately 72%, and apoB by approximately 16% versus placebo at 6 months, but did not significantly change non-calcified plaque volume compared with placebo at 12 months. No significant differences were observed for low-attenuation, calcified, or total plaque volumes [19].

This is an important negative result, but its interpretation should be precise. First, plaque regression is a demanding endpoint. Even potent LDL-lowering therapies often produce modest plaque-volume changes over short follow-up. A therapy could reduce postprandial lipemia, inflammatory activity, endothelial activation, or thrombogenicity without producing measurable regression in total plaque volume over one year.

Second, the background therapy context matters. Close to all participants were treated with any lipid-lowering agent and the average LDL-C level was 80 mg/dL (2.1 mmol/L). 78.5% were treated with statins, 23.5% with ezetimibe, 21% with fibrates, 6% with a PCSK9 inhibitor, 27.5% with omega-3 FAs and 46% had 2 or more therapies. In addition, 18% were on GLP-1 receptor agonists and 26% on SGLT2 inhibitors. Consequently, there was no plaque progression in the placebo group. If plaque progression due to LDL-lowering and other means has already been halted, TRL-lowering in this context may not lead to further plaque regression.

Third, the timing of intervention may matter. Human genetics captures lifelong differences in TRL concentration beginning before plaque develops. This study intervened when atherosclerosis is established and may be partly calcified or fibrotic. TRL lowering may be more effective at preventing plaque initiation or acceleration than at reversing established plaque, especially over a relatively short time period of 12 months.

Thus, the ESSENCE-TIMI 73b imaging study challenges a simplistic triglyceride-lowering model, but it does not refute TRL causality. It shows that potent lowering of triglycerides and TRL-C is not automatically sufficient to produce rapid structural plaque regression on top of contemporary preventive therapy.

## TRLs as Residual Risk in the Era of Intensive LDL-C Lowering

The clinical question has changed. Historically, triglycerides were studied in populations with less intensive LDL-C lowering. Today, high-risk patients may receive statins, ezetimibe, PCSK9 inhibitors, bempedoic acid, inclisiran, GLP-1 receptor agonists, SGLT2 inhibitors, and improved blood pressure treatment. In this context, the question is no longer simply whether TRLs are causal. The question is how much modifiable TRL-mediated risk remains after LDL-C/apoB, diabetes, obesity, and hypertension are treated.

This may explain why therapeutic translation is difficult. One way to conceptualize TRLs is not as an alternative to LDL, but as a modifier of apoB-driven plaque biology. LDL particles may provide the dominant bulk of cholesterol deposition within the arterial wall, whereas TRLs may act as accelerants by promoting endothelial dysfunction, inflammation, and foam-cell formation. Under this model, TRL lowering should be most effective when there is still active lipid-driven plaque progression to modify. If LDL-C and apoB remain high, lowering TRLs may have measurable benefit because the patient has ongoing apoB-mediated cholesterol deposition and substantial residual lipid risk. By contrast, if LDL-C/apoB has already been reduced to low levels, bulk cholesterol deposition may be minimized, plaque progression slowed or absent, and metabolic risk factors more optimally treated. In that setting, additional TRL lowering may yield smaller absolute benefit, or require much larger trials and longer follow-up to detect.

Thus, the absence of a large treatment effect in intensively treated populations would not necessarily refute the causal role of TRLs. Rather, it would suggest that the therapeutic relevance of TRLs is context-dependent: greatest when TRLs operate on a background of active apoB-driven atherogenesis, and smaller when the principal substrate for plaque growth has already been suppressed. This framing may help reconcile strong genetic and epidemiological evidence for TRLs with the more variable results of triglyceride-lowering intervention trials.

## Conclusions

The evidence linking TRLs to ASCVD has become increasingly compelling. Epidemiological studies show consistent associations between elevated triglycerides, TRL-related cholesterol measures, and cardiovascular risk, while human genetics strongly supports a causal role for TRLs. However, therapeutic translation has been less straightforward than for LDL-C. Fibrates have produced inconsistent results, icosapent ethyl reduces events but likely through mechanisms beyond triglyceride lowering alone, and potent APOC3 inhibition has not yet demonstrated short-term coronary plaque regression.

A key distinction is that TRLs may be causal without being equally modifiable at all stages of disease. If LDL provides the dominant bulk of arterial cholesterol deposition and TRLs act mainly as accelerants of plaque progression, the benefit of TRL lowering may depend on the presence of ongoing apoB-driven atherogenesis. In patients with high LDL-C, high apoB, and substantial TRL burden, TRL lowering may have meaningful clinical potential. In contrast, when LDL-C/apoB and metabolic risk factors are already intensively treated, the incremental benefit may be smaller and harder to demonstrate.

Thus, the central question is no longer whether TRLs are biologically relevant to ASCVD, but whether TRL-targeted therapies can reduce cardiovascular events beyond contemporary prevention, and in which patients. Future trials will need to identify the clinical contexts in which TRL lowering can potentially modify ASCVD risk.

## Key References

- Marston NA, Bergmark BA, Prohaska TA, Moura FA, Zimerman A, Alexander VJ, et al. Effect of APOC3 inhibition with olezarsen on coronary atherosclerosis: Essence-TIMI 73b imaging study. Circulation. 2026 Mar 30. 10.1161/CIRCULATIONAHA.126.080012.
  - ○ Examined the effect of Olezarsen on CCTA-quantified plaque progression and found no effect despite substantial TRL-lowering.

## Data Availability

No datasets were generated or analysed during the current study.

## References

[1] Collaboration PS, Lewington S, Whitlock G, Clarke R, Sherliker P, Emberson J, et al. Blood cholesterol and vascular mortality by age, sex, and blood pressure: a meta-analysis of individual data from 61 prospective studies with 55,000 vascular deaths. Lancet. 2007;370:1829–39. 10.1016/S0140-6736(07)61778-4. 18061058 10.1016/S0140-6736(07)61778-4

[2] Collaboration ERF, Di Angelantonio E, Sarwar N, Perry P, Kaptoge S, Ray KK, et al. Major lipids, apolipoproteins, and risk of vascular disease. JAMA. 2009;302:1993–2000. 10.1001/jama.2009.1619. 19903920 10.1001/jama.2009.1619 PMC3284229

[3] Cohen JC, Boerwinkle E, Mosley TH Jr, Hobbs HH. Sequence variations in PCSK9, low LDL, and protection against coronary heart disease. N Engl J Med. 2006;354:1264–72. 10.1056/NEJMoa054013. 16554528 10.1056/NEJMoa054013

[4] Ference BA, Yoo W, Alesh I, Mahajan N, Mirowska KK, Mewada A, et al. Effect of long-term exposure to lower low-density lipoprotein cholesterol beginning early in life on the risk of coronary heart disease: a Mendelian randomization analysis. J Am Coll Cardiol. 2012;60:2631–9. 10.1016/j.jacc.2012.09.017. 23083789 10.1016/j.jacc.2012.09.017

[5] Ference BA, Majeed F, Penumetcha R, Flack JM, Brook RD. Effect of naturally random allocation to lower low-density lipoprotein cholesterol on the risk of coronary heart disease mediated by polymorphisms in NPC1L1, HMGCR, or both: a 2 x 2 factorial Mendelian randomization study. J Am Coll Cardiol. 2015;65:1552–61. 10.1016/j.jacc.2015.02.020. 25770315 10.1016/j.jacc.2015.02.020 PMC6101243

[6] Ference BA, Ginsberg HN, Graham I, Ray KK, Packard CJ, Bruckert E, et al. Low-density lipoproteins cause atherosclerotic cardiovascular disease. Evidence from genetic, epidemiologic, and clinical studies: a consensus statement from the European Atherosclerosis Society Consensus Panel. Eur Heart J. 2017;38:2459–72. 10.1093/eurheartj/ehx144. 28444290 10.1093/eurheartj/ehx144 PMC5837225

[7] Cholesterol Treatment Trialists’ Collaboration, Baigent C, Blackwell L, Emberson J, Holland LE, Reith C, et al. Efficacy and safety of more intensive lowering of LDL cholesterol: a meta-analysis of data from 170,000 participants in 26 randomised trials. Lancet. 2010;376:1670–81. 10.1016/S0140-6736(10)61350-5. 21067804 10.1016/S0140-6736(10)61350-5 PMC2988224

[8] Cholesterol Treatment Trialists’ Collaboration, Mihaylova B, Emberson J, Blackwell L, Keech A, Simes J, et al. The effects of lowering LDL cholesterol with statin therapy in people at low risk of vascular disease: meta-analysis of individual data from 27 randomised trials. Lancet. 2012;380:581–90. 10.1016/S0140-6736(12)60367-5. 22607822 10.1016/S0140-6736(12)60367-5 PMC3437972

[9] Cannon CP, Blazing MA, Giugliano RP, McCagg A, White JA, Theroux P, et al. Ezetimibe added to statin therapy after acute coronary syndromes. N Engl J Med. 2015;372:2387–97. 10.1056/NEJMoa1410489. 26039521 10.1056/NEJMoa1410489

[10] Sabatine MS, Giugliano RP, Keech AC, Honarpour N, Wiviott SD, Murphy SA, et al. Evolocumab and clinical outcomes in patients with cardiovascular disease. N Engl J Med. 2017;376:1713–22. 10.1056/NEJMoa1615664. 28304224 10.1056/NEJMoa1615664

[11] Schwartz GG, Steg PG, Szarek M, Bhatt DL, Bittner VA, Diaz R, et al. Alirocumab and cardiovascular outcomes after acute coronary syndrome. N Engl J Med. 2018;379:2097–107. 10.1056/NEJMoa1801174. 30403574 10.1056/NEJMoa1801174

[12] Silverman MG, Ference BA, Im K, Wiviott SD, Giugliano RP, Grundy SM, et al. Association between lowering LDL-C and cardiovascular risk reduction among different therapeutic interventions: a systematic review and meta-analysis. JAMA. 2016;316:1289–97. 10.1001/jama.2016.13985. 27673306 10.1001/jama.2016.13985

[13] Khera AV, Won HH, Peloso GM, O’Dushlaine C, Liu D, Stitziel NO, et al. Association of rare and common variation in the lipoprotein lipase gene with coronary artery disease. JAMA. 2017;317:937–46. 10.1001/jama.2017.0972. 28267856 10.1001/jama.2017.0972 PMC5664181

[14] Crosby J, Peloso GM, Auer PL, Crosslin DR, Stitziel NO, Lange LA, et al. Loss-of-function mutations in APOC3, triglycerides, and coronary disease. N Engl J Med. 2014;371:22–31. 10.1056/NEJMoa1307095. 24941081 10.1056/NEJMoa1307095 PMC4180269

[15] Stitziel NO, Khera AV, Wang X, Bierhals AJ, Vourakis AC, Sperry AE, et al. ANGPTL3 deficiency and protection against coronary artery disease. J Am Coll Cardiol. 2017;69:2054–63. 10.1016/j.jacc.2017.02.030. 28385496 10.1016/j.jacc.2017.02.030 PMC5404817

[16] Dewey FE, Gusarova V, O’Dushlaine C, Gottesman O, Trejos J, Hunt C, et al. Inactivating variants in ANGPTL4 and risk of coronary artery disease. N Engl J Med. 2016;374:1123–33. 10.1056/NEJMoa1510926. 26933753 10.1056/NEJMoa1510926 PMC4900689

[17] Pradhan AD, Glynn RJ, Fruchart JC, MacFadyen JG, Zaharris ES, Everett BM, et al. Triglyceride lowering with pemafibrate to reduce cardiovascular risk. N Engl J Med. 2022;387:1923–34. 10.1056/NEJMoa2210645. 36342113 10.1056/NEJMoa2210645

[18] Bhatt DL, Steg PG, Miller M, Brinton EA, Jacobson TA, Ketchum SB, et al. Cardiovascular risk reduction with icosapent ethyl for hypertriglyceridemia. N Engl J Med. 2019;380:11–22. 10.1056/NEJMoa1812792. 30415628 10.1056/NEJMoa1812792

[19] Marston NA, Bergmark BA, Prohaska TA, Moura FA, Zimerman A, Alexander VJ, et al. Effect of APOC3 inhibition with olezarsen on coronary atherosclerosis: essence-TIMI 73b imaging study. Circulation. 2026. 10.1161/CIRCULATIONAHA.126.080012. 41910513 10.1161/CIRCULATIONAHA.126.080012

[20] Castelli WP. The triglyceride issue: a view from Framingham. Am Heart J. 1986;112:432–7. 3739899 10.1016/0002-8703(86)90296-6

[21] Austin MA. Plasma triglyceride and coronary heart disease. Arterioscler Thromb. 1991;11:2–14. 1987999 10.1161/01.atv.11.1.2

[22] Castelli WP, Anderson K. A population at risk: prevalence of high cholesterol levels in hypertensive patients in the Framingham Study. Am J Med. 1986;80:23–32. 3946458 10.1016/0002-9343(86)90157-9

[23] Hokanson JE, Austin MA. Plasma triglyceride level is a risk factor for cardiovascular disease independent of high-density lipoprotein cholesterol level: a meta-analysis of population-based prospective studies. J Cardiovasc Risk. 1996;3:213–9. 8836866

[24] Sarwar N, Danesh J, Eiriksdottir G, Sigurdsson G, Wareham N, Bingham S, et al. Triglycerides and the risk of coronary heart disease: 10,158 incident cases among 262,525 participants in 29 Western prospective studies. Circ. 2007;115:450–8. 10.1161/CIRCULATIONAHA.106.637793. 10.1161/CIRCULATIONAHA.106.637793 17190864

[25] Patel A, Barzi F, Jamrozik K, Lam TH, Ueshima H, Whitlock G, et al. Serum triglycerides as a risk factor for cardiovascular diseases in the Asia-Pacific region. Circulation. 2004;110:2678–86. 10.1161/01.CIR.0000145615.33955.83. 15492305 10.1161/01.CIR.0000145615.33955.83

[26] Sharrett AR, Ballantyne CM, Coady SA, Heiss G, Sorlie PD, Catellier D, et al. Coronary heart disease prediction from lipoprotein cholesterol levels, triglycerides, lipoprotein(a), apolipoproteins A-I and B, and HDL density subfractions: the Atherosclerosis Risk in Communities Study. Circulation. 2001;104:1108–13. 10.1161/hc3501.095214. 11535564 10.1161/hc3501.095214

[27] Nordestgaard BG, Benn M, Schnohr P, Tybjaerg-Hansen A. Nonfasting triglycerides and risk of myocardial infarction, ischemic heart disease, and death in men and women. JAMA. 2007;298:299–308. 10.1001/jama.298.3.299. 17635890 10.1001/jama.298.3.299

[28] Bansal S, Buring JE, Rifai N, Mora S, Sacks FM, Ridker PM. Fasting compared with nonfasting triglycerides and risk of cardiovascular events in women. JAMA. 2007;298:309–16. 10.1001/jama.298.3.309. 17635891 10.1001/jama.298.3.309

[29] Freiberg JJ, Tybjaerg-Hansen A, Jensen JS, Nordestgaard BG. Nonfasting triglycerides and risk of ischemic stroke in the general population. JAMA. 2008;300:2142–52. 10.1001/jama.2008.621. 19001625 10.1001/jama.2008.621

[30] Varbo A, Nordestgaard BG, Tybjaerg-Hansen A, Schnohr P, Jensen GB, Benn M. Nonfasting triglycerides, cholesterol, and ischemic stroke in the general population. Ann Neurol. 2011;69:628–34. 10.1002/ana.22384. 21337605 10.1002/ana.22384

[31] Varbo A, Benn M, Tybjaerg-Hansen A, Jorgensen AB, Frikke-Schmidt R, Nordestgaard BG. Remnant cholesterol as a causal risk factor for ischemic heart disease. J Am Coll Cardiol. 2013;61:427–36. 10.1016/j.jacc.2012.08.1026. 23265341 10.1016/j.jacc.2012.08.1026

[32] Varbo A, Benn M, Tybjaerg-Hansen A, Nordestgaard BG. Elevated remnant cholesterol causes both low-grade inflammation and ischemic heart disease, whereas elevated low-density lipoprotein cholesterol causes ischemic heart disease without inflammation. Circulation. 2013;128:1298–309. 10.1161/CIRCULATIONAHA.113.003008. 23926208 10.1161/CIRCULATIONAHA.113.003008

[33] Varbo A, Freiberg JJ, Nordestgaard BG. Remnant cholesterol and myocardial infarction in normal weight, overweight, and obese individuals from the Copenhagen General Population Study. Clin Chem. 2018;64:219–30. 10.1373/clinchem.2017.279463. 29021326 10.1373/clinchem.2017.279463

[34] Jepsen AMK, Langsted A, Varbo A, Bang LE, Kamstrup PR, Nordestgaard BG. Increased remnant cholesterol explains part of residual risk of all-cause mortality in 5414 patients with ischemic heart disease. Clin Chem. 2016;62:593–604. 10.1373/clinchem.2015.253757. 26888894 10.1373/clinchem.2015.253757

[35] Liu J, Zeng FF, Liu ZM, Zhang CX, Ling WH, Chen YM. Effects of blood triglycerides on cardiovascular and all-cause mortality: a systematic review and meta-analysis of 61 prospective studies. Lipids Health Dis. 2013;12:159. 10.1186/1476-511X-12-159. 24164719 10.1186/1476-511X-12-159 PMC4231478

[36] Do R, Willer CJ, Schmidt EM, Sengupta S, Gao C, Peloso GM, et al. Common variants associated with plasma triglycerides and risk for coronary artery disease. Nat Genet. 2013;45:1345–52. 10.1038/ng.2795. 24097064 10.1038/ng.2795 PMC3904346

[37] Jorgensen AB, Frikke-Schmidt R, Nordestgaard BG, Tybjaerg-Hansen A. Loss-of-function mutations in APOC3 and risk of ischemic vascular disease. N Engl J Med. 2014;371:32–41. 10.1056/NEJMoa1308027. 24941082 10.1056/NEJMoa1308027

[38] Ference BA, Kastelein JJP, Ray KK, Ginsberg HN, Chapman MJ, Packard CJ, et al. Association of triglyceride-lowering LPL variants and LDL-C-lowering LDLR variants with risk of coronary heart disease. JAMA. 2019;321:364–73. 10.1001/jama.2018.20045. 30694319 10.1001/jama.2018.20045 PMC6439767

[39] Richardson TG, Sanderson E, Palmer TM, Ala-Korpela M, Ference BA, Davey Smith G, et al. Evaluating the relationship between circulating lipoprotein lipids and apolipoproteins with risk of coronary heart disease: a multivariable Mendelian randomisation analysis. PLoS Med. 2020;17:e1003062. 10.1371/journal.pmed.1003062. 32203549 10.1371/journal.pmed.1003062 PMC7089422

[40] Helgadottir A, Gretarsdottir S, Thorleifsson G, Hjartarson E, Sigurdsson A, Magnusdottir A, et al. Variants with large effects on blood lipids and the role of cholesterol and triglycerides in coronary disease. Nat Genet. 2016;48:634–9. 10.1038/ng.3561. 27135400 10.1038/ng.3561 PMC9136713

[41] Björnson E, Adiels M, Taskinen MR, Burgess S, Rawshani A, Boren J, et al. Triglyceride-rich lipoprotein remnants, low-density lipoproteins, and risk of coronary heart disease: a UK Biobank study. Eur Heart J. 2023;44:4186–95. 10.1093/eurheartj/ehad337. 37358553 10.1093/eurheartj/ehad337 PMC10576615

[42] Björnson E, Adiels M, Gummesson A, Taskinen MR, Burgess S, Packard CJ, et al. Quantifying triglyceride-rich lipoprotein atherogenicity, associations with inflammation, and implications for risk assessment using non-HDL cholesterol. J Am Coll Cardiol. 2024;84:1328–38. 10.1016/j.jacc.2024.07.034. 39322327 10.1016/j.jacc.2024.07.034 PMC7616757

[43] Boren J, Williams KJ. The central role of arterial retention of cholesterol-rich apolipoprotein-B-containing lipoproteins in the pathogenesis of atherosclerosis: a triumph of simplicity. Curr Opin Lipidol. 2016;27:473–83. 10.1097/MOL.0000000000000330. 27472409 10.1097/MOL.0000000000000330

[44] Ference BA, Graham I, Tokgozoglu L, Catapano AL. Impact of lipids on cardiovascular health: JACC Health Promotion Series. J Am Coll Cardiol. 2018;72:1141–56. 10.1016/j.jacc.2018.06.046. 30165986 10.1016/j.jacc.2018.06.046

[45] Chapman MJ, Packard CJ, Björnson E, Ginsberg HN, Borén J. Triglyceride-rich lipoproteins, remnants and atherosclerotic cardiovascular disease: What we know and what we need to know. Atherosclerosis. 2025;410:120529. 10.1016/j.atherosclerosis.2025.120529. 41202476 10.1016/j.atherosclerosis.2025.120529

[46] Kraaijenhof JM, Hovingh GK, Stroes ESG, Kroon J. The iterative lipid impact on inflammation in atherosclerosis. Curr Opin Lipidol. 2021;32:286–92. 10.1097/MOL.0000000000000779. 34392272 10.1097/MOL.0000000000000779 PMC8452331

[47] Elias-Lopez D, Doi T, Nordestgaard BG, Kobylecki CJ. Remnant cholesterol and low-grade inflammation jointly in atherosclerotic cardiovascular disease: implications for clinical trials. Curr Opin Clin Nutr Metab Care. 2024;27:125–35. 10.1097/MCO.0000000000000999. 38320159 10.1097/MCO.0000000000000999

[48] Elias-Lopez D, Kobylecki CJ, Vedel-Krogh S, Doi T, Nordestgaard BG. Association of low-grade inflammation and elevated remnant cholesterol with risk of ASCVD and mortality in impaired renal function. Atherosclerosis. 2025;406:119241. 10.1016/j.atherosclerosis.2025.119241. 40435574 10.1016/j.atherosclerosis.2025.119241

[49] Morze J, Björnson E, Mi MY, Adiels M, Melloni GE, Rosas da Silva G, et al. Plasma proteomic profiling reveals distinct roles of apolipoprotein B-containing lipoproteins in atherosclerosis. Res Square [Preprint]. 2025 Dec;19. 10.21203/rs.3.rs-8307321/v1.

[50] Frick MH, Elo O, Haapa K, Heinonen OP, Heinsalmi P, Helo P, et al. Helsinki heart study: primary-prevention trial with gemfibrozil in middle-aged men with dyslipidemia. N Engl J Med. 1987;317:1237–45. 10.1056/NEJM198711123172001. 3313041 10.1056/NEJM198711123172001

[51] Rubins HB, Robins SJ, Collins D, Fye CL, Anderson JW, Elam MB, et al. Gemfibrozil for the secondary prevention of coronary heart disease in men with low levels of high-density lipoprotein cholesterol. N Engl J Med. 1999;341:410–8. 10.1056/NEJM199908053410604. 10438259 10.1056/NEJM199908053410604

[52] Keech A, Simes RJ, Barter P, Best J, Scott R, Taskinen MR, et al. Effects of long-term fenofibrate therapy on cardiovascular events in 9795 people with type 2 diabetes mellitus: the FIELD study. Lancet. 2005;366:1849–61. 10.1016/S0140-6736(05)67667-2. 16310551 10.1016/S0140-6736(05)67667-2

[53] ACCORD Study Group, Ginsberg HN, Elam MB, Lovato LC, Crouse JR III, Leiter LA, et al. Effects of combination lipid therapy in type 2 diabetes mellitus. N Engl J Med. 2010;362:1563–74. 10.1056/NEJMoa1001282. 20228404 10.1056/NEJMoa1001282 PMC2879499

[54] Investigators ORIGINT, Bosch J, Gerstein HC, Dagenais GR, Diaz R, Dyal L, et al. n-3 fatty acids and cardiovascular outcomes in patients with dysglycemia. N Engl J Med. 2012;367:309–18. 10.1056/NEJMoa1203859. 22686415 10.1056/NEJMoa1203859

[55] ASCEND Study Collaborative Group, Bowman L, Mafham M, Wallendszus K, Stevens W, Buck G, et al. Effects of n-3 fatty acid supplements in diabetes mellitus. N Engl J Med. 2018;379:1540–50. 10.1056/NEJMoa1804989. 30146932 10.1056/NEJMoa1804989

[56] Nicholls SJ, Lincoff AM, Garcia M, Bash D, Ballantyne CM, Barter PJ, et al. Effect of high-dose omega-3 fatty acids versus corn oil on major adverse cardiovascular events in patients at high cardiovascular risk: the STRENGTH randomized clinical trial. JAMA. 2020;324:2268–80. 10.1001/jama.2020.22258. 33190147 10.1001/jama.2020.22258 PMC7667577

[57] Budoff MJ, Bhatt DL, Kinninger A, Lakshmanan S, Muhlestein JB, Le VT, et al. Effect of icosapent ethyl on progression of coronary atherosclerosis in patients with elevated triglycerides on statin therapy: final results of the EVAPORATE trial. Eur Heart J. 2020;41:3925–32. 10.1093/eurheartj/ehaa652. 32860032 10.1093/eurheartj/ehaa652 PMC7654934

[58] Hu Y, Hu FB, Manson JE. Marine omega-3 supplementation and cardiovascular disease: an updated meta-analysis of 13 randomized controlled trials involving 127,477 participants. J Am Heart Assoc. 2019;8:e013543. 10.1161/JAHA.119.013543. 31567003 10.1161/JAHA.119.013543 PMC6806028

[59] Witztum JL, Gaudet D, Freedman SD, Alexander VJ, Digenio A, Williams KR, et al. Volanesorsen and triglyceride levels in familial chylomicronemia syndrome. N Engl J Med. 2019;381:531–42. 10.1056/NEJMoa1715944. 31390500 10.1056/NEJMoa1715944

[60] Bergmark BA, Marston NA, Prohaska TA, Alexander VJ, Zimerman A, Moura FA, et al. Targeting APOC3 with olezarsen in moderate hypertriglyceridemia. N Engl J Med. 2025;393:1279–91. 10.1056/NEJMoa2507227. 40888739 10.1056/NEJMoa2507227

[61] Ballantyne CM, Vasas S, Azizad M, Clifton P, Rosenson RS, Chang T, et al. Plozasiran, an RNA interference agent targeting APOC3, for mixed hyperlipidemia. N Engl J Med. 2024;391:899–912. 10.1056/NEJMoa2404143. 38804517 10.1056/NEJMoa2404143

[62] Gaudet D, Pall D, Watts GF, Nicholls SJ, Rosenson RS, Modesto K, et al. Plozasiran (ARO-APOC3) for severe hypertriglyceridemia: the SHASTA-2 randomized clinical trial. JAMA Cardiol. 2024;9:620–30. 10.1001/jamacardio.2024.0959. 38583092 10.1001/jamacardio.2024.0959 PMC11000138

[63] Masson W, Lobo M, Nogueira JP, Corral P, Barbagelata L, Siniawski D. Inhibitors of apolipoprotein C3, triglyceride levels, and risk of pancreatitis: a systematic review and meta-analysis. Rev Endocr Metab Disord. 2024;25:817–25. 10.1007/s11154-024-09893-x. 38997541 10.1007/s11154-024-09893-x

[64] Raal FJ, Rosenson RS, Reeskamp LF, Hovingh GK, Kastelein JJP, Rubba P, et al. Evinacumab for homozygous familial hypercholesterolemia. N Engl J Med. 2020;383:711–20. 10.1056/NEJMoa2004215. 32813947 10.1056/NEJMoa2004215

[65] Ahmad Z, Pordy R, Rader DJ, Rosenson RS, Genest J, Leiter LA, et al. Inhibition of angiopoietin-like protein 3 with evinacumab in subjects with high and severe hypertriglyceridemia. J Am Coll Cardiol. 2021;78:193–5. 10.1016/j.jacc.2021.04.091. 34238441 10.1016/j.jacc.2021.04.091

[66] Bergmark BA, O’Donoghue ML, Murphy SA, Kuder JF, Ezhov MV, Ceska R, et al. Effect of vupanorsen on non-high-density lipoprotein cholesterol levels in statin-treated patients with elevated cholesterol: TRANSLATE-TIMI 70. Circulation. 2022;145:1377–86. 10.1161/CIRCULATIONAHA.122.059266. 35369705 10.1161/CIRCULATIONAHA.122.059266 PMC9047643

